# Reversible Myoclonus‐Ataxia as a Postinfectious Manifestation of COVID‐19

**DOI:** 10.1002/mdc3.13088

**Published:** 2020-09-28

**Authors:** Mijntje M.I. Schellekens, Chantal P. Bleeker‐Rovers, Pim A.J. Keurlings, Catherine J. Mummery, Bastiaan R. Bloem

**Affiliations:** ^1^ Department of Neurology, Centre of Expertise for Parkinson & Movement Disorders, Donders Institute for Brain, Cognition and Behavior Radboud University Medical Centre Nijmegen The Netherlands; ^2^ Department of Internal Medicine, Division of Infectious Diseases Radboud University Medical Centre Nijmegen The Netherlands; ^3^ Department of Primary and Community Care Radboud University Medical Centre Nijmegen The Netherlands; ^4^ Dementia Research Centre National Hospital for Neurology and Neurosurgery London United Kingdom

**Keywords:** COVID‐19, severe acute respiratory syndrome coronavirus 2 (SARS‐CoV‐2), myoclonus, ataxia, postinfectious

The new severe acute respiratory syndrome coronavirus 2 (SARS‐CoV‐2) has led to the coronavirus disease 2019 (COVID‐19) in many people worldwide. COVID‐19 features a wide spectrum of symptoms, including neurological manifestations.^1^, ^2^, ^3^, ^4^, ^5^ A recent report drew attention to generalized myoclonus in hospitalized COVID‐19 patients, who typically had severe respiratory symptoms necessitating intensive care unit admission.^6^ Suspecting a postinfectious etiology, all of these patients received immunotherapy. Here we describe a patient with COVID‐19 and myoclonus combined with cerebellar ataxia who was managed entirely on an outpatient basis and who recovered spontaneously.

## Case Report

A 48‐year‐old man with a history of asymptomatic HIV infection, with normal CD‐4 count and undetectable viral load, for which he received treatment with antiviral therapy (dolutegravir, abacavir, lamivudine) was referred to the outpatient clinic of the Radboud University Medical Centre because of the onset of jerky movements that had emerged after the resolution of symptoms related to COVID‐19. His only other medication was pantoprazole. On day 1, this patient developed a fever and a concomitant headache but with minimal cough. On day 7, the general practitioner treated him with amoxicillin for 5 days. Symptoms gradually resolved except for persistent fatigue. On day 13, he developed involuntary jerky movements and clumsiness of his trunk and limbs during the course of several hours. The jerks were present at rest but worsened with movement, causing considerable disability. Gait had become insecure. Following the initial subacute onset, his complaints did not progress any further. A nasopharyngeal swab tested positive for SARS‐CoV‐2 by polymerase chain reaction (PCR on day 16). PCR was not repeated.

Neurological examination on day 20 revealed a normal consciousness with fully intact cognitive functions. Eye movements showed saccadic intrusions and hypermetric saccades, but no opsoclonus. There was facial jerking. Examination of the cranial nerves was otherwise unremarkable. There were generalized myoclonic jerks of the trunk and limbs, particularly involving the hands, which were present at rest but that clearly worsened both posturally and with action (Video SS1). The myoclonus was not stimulus sensitive to touch, and there was no startle response to a loud acoustic stimulus. Except for some lapses in the legs upon rising from a chair, there was no convincing negative myoclonus. Motor examination further revealed clear cerebellar ataxia of the arms and legs (hypermetria) and an ataxic gait (Video SS1). There was no paresis or sensory deficit. Deep tendon reflexes were normal, and plantar responses were unremarkable.

An extensive laboratory work‐up, including thyroid function test and celiac disease screening, cranial magnetic resonance imaging (MRI) with gadolinium, and extensive cerebrospinal fluid (CSF) analysis, revealed no abnormalities (Table 1). PCR for SARS‐CoV‐2 in CSF on day 35 was negative. SARS‐CoV‐2 serology on day 62 was positive.

**TABLE 1:** *Complementary tests*

Laboratory findings	Results	Day of illness
C‐reactive protein (mg/L)	<1	35
Glucose (mmol/L)	5.4	35
TSH (mE/L)	1.49	35
Celiac disease screening	Negative	35
Syphilis screening	Negative	35
Anti‐VGKC	Negative	35
SARS‐CoV‐2 IgG	Positive	62
PCR SARS‐CoV‐2 (nasopharyngeal swab)	Positive	13
CSF analyses		
Leucocytes (/μL)	2	35
Glucose (mmol/L)	3.4	35
Protein (mg/L)	286	35
Isoelectric focusing	Normal	35
PCR SARS‐CoV‐2	Negative	35
PCR enterovirus, HSV1, HSV2, VZV, mycoplasma	Negative	35
PCR HIV load	Below the quantification limit	35
Paraneoplastic antibodies	Negative	35
Brain MRI scan (with gadolinium)	Normal	27

Abbreviations: TSH, thyroid‐stimulating hormone; VGKC, voltage‐gated potassium channel; SARS‐CoV‐2, severe acute respiratory syndrome coronavirus 2; IgG, immunoglobulin G; PCR, polymerase chain reaction; CSF, cerebrospinal fluid; HSV1, herpes simplex virus 1; HSV2, herpes simplex virus 2; VZV, varicella zoster virus; MRI, magnetic resonance imaging.

No immunotherapy was given, but symptomatic treatment with levetiracetam was initiated, which alleviated the myoclonus within several days. Functional disability also diminished considerably. Repeat neurological examination several weeks later (day 62) showed that both the myoclonus and the ataxia had improved, but recovery was not yet complete (Video SS2).

## Discussion

This is the first reported case of myoclonus‐ataxia in a patient with COVID‐19 who was not admitted to a hospital. Extensive evaluation, including blood tests, brain MRI, and CSF analyses, excluded other causes for the presentation. A metabolic cause seemed unlikely in light of the temporal course and the normal brain MRI. The subacute onset of symptoms after the disappearance of the typical clinical manifestations of COVID‐19, in the absence of any other identifiable cause, leads us to postulate a postinfectious immune‐mediated etiology. A direct effect of COVID‐19 seems much less likely given the relatively late onset of the myoclonus‐ataxia in the disease course and considering the unremarkable results of the cerebral MRI and CSF. Moreover, movement disorders including ataxia and myoclonus have been described as a postinfectious manifestation following other virus infections.^7^, ^8^

Generalized myoclonus following COVID‐19 has been described previously in 3 patients,^6^ but the present case history offers a clinically relevant expansion of the syndrome. First, our patient demonstrated not only generalized myoclonus but also clear signs of cerebellar ataxia. In addition, our patient was not admitted to a hospital because the respiratory manifestations were only mild, hence postinfectious syndromes are not just a feature of a severe systemic disease course. Unlike the published cases, this patient did not have hypersomnolence or other features of encephalopathy. Therefore, we recommend considering COVID‐19 as a possible cause of myoclonus‐ataxia, or another movement disorder in this spectrum, also in patients with only mild, even unrecognized, typical COVID‐19 manifestations. We considered that the mild initial presentation of the COVID‐19 infection and the mostly postinfectious symptoms arose because this patient was on antiviral treatment for HIV. Patients with HIV on antiviral treatment may be at decreased risk for COVID‐19 because this medication may have activity against coronaviruses,^9^ so it is conceivable that antiretroviral treatment also affects the presentation of COVID‐19. More research is needed to understand this. Finally, our patient did not receive immunotherapy and started to recover spontaneously over time, so depending on the severity of the clinical manifestations, only symptomatic treatment may be considered in ambulatory cases.

## Author Roles

(1) Research Project: A. Conception; (2) Manuscript Preparation: A. Writing of the First Draft, B. Review and Critique.

M.M.I.S.: 1A, 2A

C.P.B.‐R.: 2B

P.A.J.K.: 2B

C.J.M.: 2B

B.R.B.: 1A, 2B

## Disclosures

**Ethical Compliance Statement:** The authors confirm that the approval of an institutional review board was not required for this work. Written and oral informed consent was obtained to record and use these videos for the present publication. We confirm that we have read the Journal's position on issues involved in ethical publication and affirm that this work is consistent with those guidelines.

**Funding Sources and Conflict of Interest:** No specific funding was received for this work. The authors declare that there are no conflicts of interest relevant to this work.

**Financial Disclosures for the Previous 12 Months:** Bas Bloem was supported by a Centre of Excellence Grant of the Parkinson's Foundation. Catherine Mummery was supported by the National Institute for Health Research University College London Hospitals Biomedical Research Centre. The other authors have no disclosures to report.

## Supporting information


**Video S1.** Generalized myoclonic jerks of the trunk and limbs, particularly involving the hands, at rest, posturally, and with action. The patient also manifests cerebellar ataxia of the arms and legs (hypermetria) and an ataxic gait. Upon standing up from the chair, a couple of lapses in the legs can be noted, but the remainder of the neurological examination showed no convincing negative myoclonus. The facial jerking cannot be seen here because of the facial mask, but became apparent in a subsequent video made by the patient at home.

**Video S2.** Improved but persistent myoclonic jerks, cerebellar ataxia, and ataxic gait.
